# Hydrothermal Synthesis of Silicoaluminophosphate with AEL Structure Using a Residue of Fluorescent Lamps as Starting Material

**DOI:** 10.3390/molecules26237366

**Published:** 2021-12-04

**Authors:** Gidiângela C. C. S. Lima, Mariele I. S. Mello, Lindiane Bieseki, Antonio S. Araujo, Sibele B. C. Pergher

**Affiliations:** 1 Molecular Sieves Laboratory (LABPEMOL), Instituto of Cheistry (IQ), Federal University of Rio Grande do Norte (UFRN), Natal 59078-970, RN, Brazil; gidiangelalima@gmail.com (G.C.C.S.L.); mellomariele@gmail.com (M.I.S.M.); lindiane.bieseki@gmail.com (L.B.); 2 Institute of Chemistry (IQ), Federal University of Rio Grande do Norte (UFRN), Natal 59078-970, RN, Brazil; antonio.araujo@ufrn.br

**Keywords:** fluorescent lamp, phosphor residue, SAPO-11, hydrothermal synthesis

## Abstract

Silicoaluminophosphate molecular sieves of SAPO-11 type (AEL structure) were synthesized by the hydrothermal method, from the residue of a fluorescent lamp as a source or Si, Al, and P in the presence of water and di-propyamine (DPA) as an organic template. To adjust the P_2_O_5_/SiO_2_ and Si/Al and ratios, specific amounts of silica, alumina, or alumina hydroxide and orthophosphoric acid were added to obtain a gel with molar chemical composition 1.0 Al_2_O_3_:1.0 P_2_O_5_:1.2 DPA:0.3 SiO_2_:120 H_2_O. The syntheses were carried out at a temperature of 473 K at crystallization times of 24, 48, and 72 h. The fluorescent lamp residue and the obtained samples were characterized by X-ray fluorescence, X-ray diffraction, scanning electron microscopy, and BET surface area analysis using nitrogen adsorption isotherms. The presence of fluorapatite was detected as the main crystalline phase in the residue, jointly with considered amounts of silica, alumina, and phosphorus in oxide forms. The SAPO-11 prepared using aluminum hydroxide as Al source, P_2_O_5_/SiO_2_ molar ratio of 3.6 and Si/Al ratio of 0.14, at crystallization time of 72 h, achieves a yield of 75% with a surface area of 113 m^2^/g, showing that the residue from a fluorescent lamp is an alternative source for development of new materials based on Si, Al, and P.

## 1. Introduction

Fluorescent lamps are widely used materials in society and consumption. However, inappropriate disposal causes environmental impacts by the presence of heavy metals such as mercury. Since 2011, more than 4800 million fluorescent lamps have been used [1]. In 2017, Brazil had already sold about 290 million fluorescent lamps per year [2]; however, little is done to prevent their waste from becoming an environmental problem. Some countries, such as Canada and the United Kingdom, provide information on fluorescent lamps’ use, production, and recycling [3,4]. In addition, many research studies have been carried out on recycling this residue, aiming at the use of glass [5,6] and separation of mercury [7,8,9,10,11].

Several research studies have been reported on the separation and speciation of transition metals from fluorescent lamp residues [12,13,14,15,16,17,18,19,20], highlighting the separation of rare earths using ionic liquids as extraction agents [13], centrifugation [14], and flotation [15]. However, research on applying these residues to obtain molecular sieves based on silicoaluminophosphates was not verified. Molecular sieve is the term used to describe porous materials based on aluminosilicates (zeolites), aluminophosphates (AlPOs), and silicoaluminophosphates (SAPOs) with different structures and specific pore sizes. These materials have an impact on industrial applications as adsorbents and catalysts [21]. For example, crystalline molecular sieves with properties similar to zeolites are known as promising catalysts for the oil industry [22,23,24,25,26,27], emphasizing the application of SAPO-34 [26,28,29] and SAPO-11 [29,30,31,32,33,34,35].

In order to prepare eco-friendly porous materials, the current work deals with the development of an alternative methodology to synthesize the SAPO-11 molecular sieve, using a residue of fluorescent lamp as an inorganic source of silicon, aluminum, and phosphorus. The crystallographic, structural, morphological, and adsorption properties of the obtained materials were evaluated.

## 2. Materials and Methods

### 2.1. Hydrothermal Synthesis of Materials

The silicoaluminophosphate SAPO-11 was synthesized according to the procedure previously reported [36] with some modifications (such as different aluminum sources) in order to obtain a gel with the molar chemical composition 1.0 Al_2_O_3_:1.0 P_2_O_5_:1.2 DPA:0.3 SiO_2_:120 H_2_O. The fluorescent lamp residue was used as additional sources of Si, Al, and P.

A typical synthesis procedure of SAPO-11 was performed as follows: the aluminum oxide (1.99 g) or aluminum hydroxide (Aldrich 76.5%–1.54 g) was first hydrolyzed in deionized water under stirring for 0.5 h. Next, 4.13 g of the orthophosphoric acid (Merck 85%) was slowly added to the mixture and homogenized for 2 h. Then, 1.14 g of the tetraethyl orthosilicate (TEOS, Aldrich, 98%) was added to the mixture and homogenized for 2 h. After this time, dipropylamine (DPA, Aldrich > 99%–2.19 g) was added to the mixture and homogenized for two additional hours, under continuous stirring.

The obtained synthesis gel was transferred into a Teflon-lined stainless-steel autoclave and heated at 473 K for times varying from 2 to 72 h. The resultant product was filtered, washed with deionized water, and dried at 373 K. The sample was calcined at 523 K for 2 h and then submitted at 923 K, with a heating rate of 8 °C/min, for an additional period of 5 h. The synthesis conditions used are given in Table 1, where 6, 12, 24, 48, and 72 are the crystallization times in hours; S means SAPO-11; A and H are aluminum oxide and aluminum hydroxide, respectively; R is the phosphor residue; and 0.5, 1, and 2 are the amount in grams of residue used. When the lamp residue is used, it provides Al, Si, and P; to achieve the same gel chemical molar composition, more Al, Si, and P are added. For example, for the 24/SHR0.5 synthesis, the following was used: 0.5 g of lamp residue, 0.22 g of TEOS, 0.8 g of Al(OH)_3_, 1.68 g of H_3_PO_4_, and 1.1 g of DPA.

### 2.2. Physicochemical Characterization

The elemental composition was determined from X-ray fluorescence (XRF) using Bruker S2Ranger equipment coupled with a Pd radiation source at 50 W, 40 kV, and 2 mA, XFlash^®^ Silicon Drift Detector. The crystallinity of the samples was followed by powder X-ray diffraction (XRD) with a Bruker D2 Phaser with monochromatic CuKalpha radiation, and the collection of the signals was carried out in a Lynxeye detector. The samples selected for phase quantification were again analyzed under conditions that allowed suitable data acquisition for refinement using the Rietveld method. The samples were also analyzed in a D2 Phaser—Bruker AXS Bragg–Brentano diffractometer operating at a voltage of 30 kV and a current of 10 mA. The analysis range was 2Θ = 2–70°, with a step size of 0.01° and a counting time of 0.3 s. Phase identification was performed using powder direction files from the International Center for Diffraction Data—ICDD end Crystallography Open Database (COD) and the crystallographic cards of zeolitic structures obtained from the International Zeolite Association (http://www.iza-online.org, accessed on 20 September 2021).

After identifying the phases, the quantitative analysis was made using the Rietveld method with TOPAS v5.0 software. The parameters that were refined were scale factors for all phases, zero-shift parameter, background using Chebychev polynomial and the 1/X Bkg function, half-width parameters, atomic site occupancies, atomic coordinates, and preferred orientation. The goodness of fit of the calculated and observed profiles was followed by the Rp, Rwp, Rexp, and the goodness of fit index (GOF) [37].

The characterization from scanning electron microscopy (SEM) was carried out using a ZEISS branded Auriga microscope with field emission gun (FEG) type emitter.

The BET surface area and pore volume were measured by N_2_ adsorption and desorption in a Micromeritics ASAP 2020 from Micromeritics. Before the measurements, all samples were outgassed under vacuum for 9 h at 473 K. The specific area was determined using the BET method. The samples’ external surface area (S_ext_) and micropore volume (V_micro_) were determined using t-plot analysis. The total pore volumes were estimated using the BJH method at *p*/*p*_0_ = 0.98 and the mesopore volumes (V_meso_) were calculated by subtracting the micropore volume (V_micro_) from the total pore volume.

## 3. Results and Discussion

### 3.1. Characterization from XRF, XRD, and SEM

**Figure 1:**
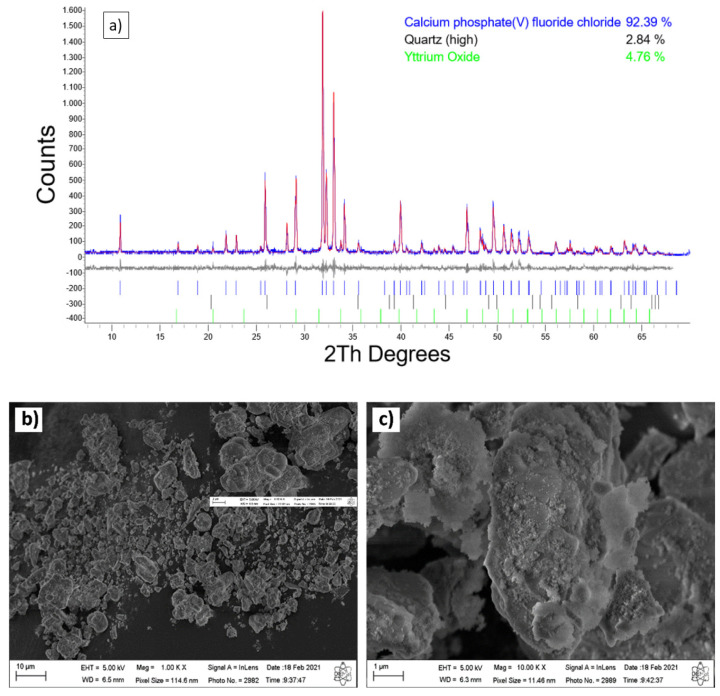
(**a**) XRD patterns of the fluorescent lamp, Rietveld refinement plot for phosphor powder, the vertical lines provide the positions of all possible Bragg reflections for the calcium phosphate (V) fluoride chloride phases (database_code-ICSD 203026), quartz (high) (database code_ICSD 89287), and yttrium oxide (database_code_ICSD 66730). The values of the standard agreement indices are Rp—12.15, Rwp—16.11, Rexp—13.74, Gof—1.17; (**b**,**c**) SEM images of the phosphor powder.

The XRD patterns of the residue of the fluorescent lamp are shown in Figure 1. The XRD pattern shows adjustment difference between experimental data (black line) and refined data (red line) obtained after refinement using the Rietveld method. Phosphor powder’s residue is composed of a mixture of three crystalline phases, 92.39% of calcium phosphate (V) fluoride chloride, 2.84% of quartz (high), and 4.76% yttrium oxide: proving to be a crystalline material. The morphology of the particles of the residues, as analyzed by SEM (Figure 1b,c), showed irregular shapes with clustered blocks. From XRF, the amounts of SiO_2_, Al_2_O_3,_ and P_2_O_5_ in the residue were 9.98, 8.90, and 27.70% in mass, respectively, which are good ratios for the SAPO-11 synthesis (Table 2). Due to the high concentration of phosphorous in the residue, it is also known as phosphor powder.

**Figure 2:**
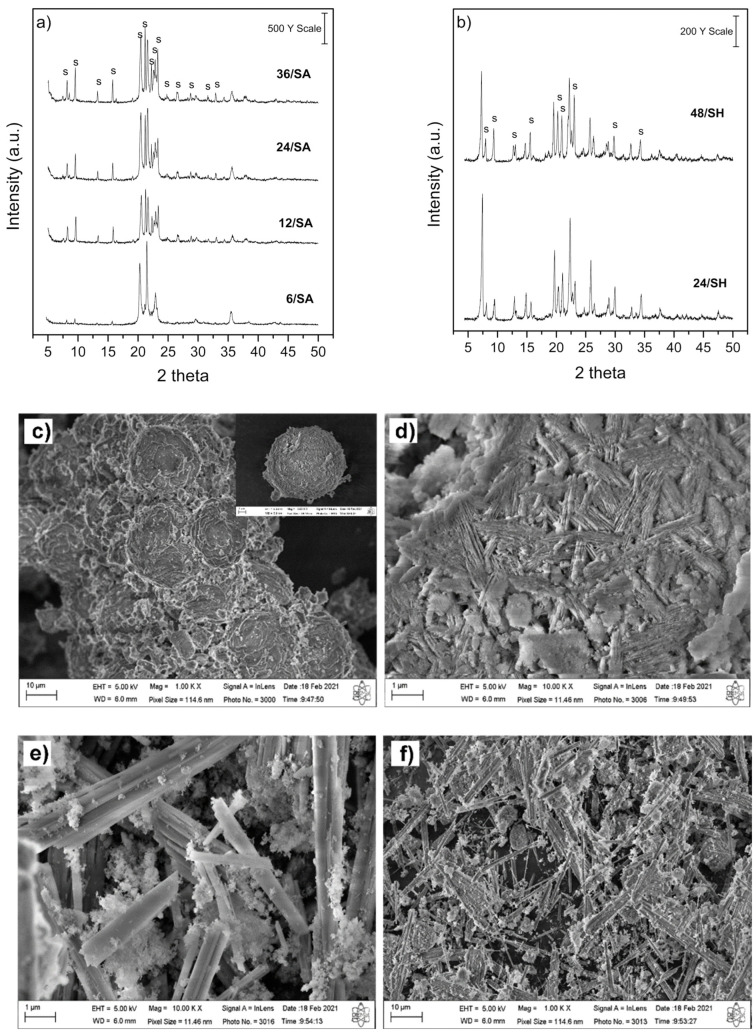
XRD patterns of the samples with different crystallization times: (**a**) SAPO-11 using aluminum oxide; (**b**) SAPO-11 using aluminum hydroxide as Al source. SEM images of (**c**,**d**) 24/SA and (**e**,**f**) 24/SH. (S) SAPO-11.

**Figure A1:**
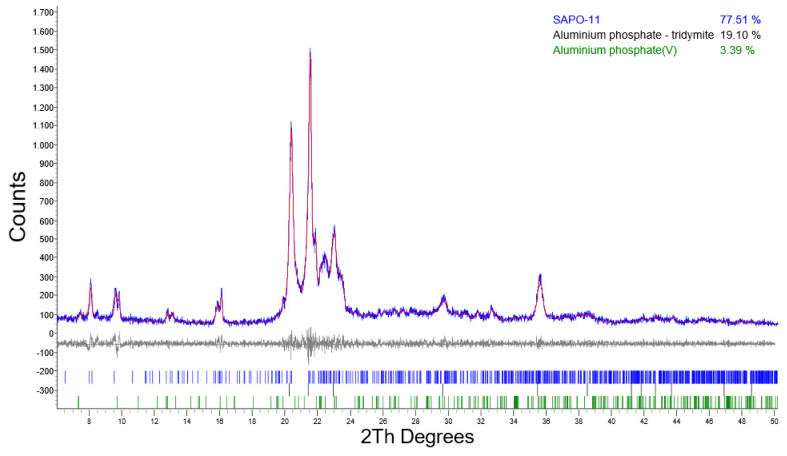
Rietveld refinement plot for 24/SA: the vertical lines provide the positions of all possible Bragg reflections for aluminum phosphate tridymite phases, according to data presented in the literature [55]. Aluminum phosphate (V) (database_code_ICSD 88566) e SAPO-11 [56]. The values of the standard agreement indices are Rp—7.51, Rwp—10.02, Rexp—9.16, GofF—1.09.

**Figure A2:**
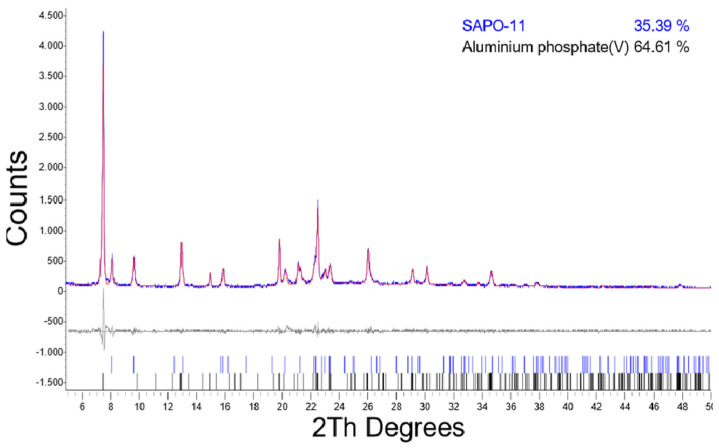
Rietveld refinement plot for 24/SH: the vertical lines provide the positions of all possible Bragg reflections for the aluminum phosphate (V) phases (database_code-ICSD 88566) and structure of zeolite type AEL (database_IZA). The values of the standard agreement indices are Rp—8.58, Rwp—11.22, Rexp—8.35, GofF—1.34.

Figure 2 shows the XRD patterns of the SAPO-11 samples using conventional methodology [36]. Figure 2a shows the XRD profiles using aluminum oxide as an Al source, varying the time from 6 to 36 h (6/SA, 12/SA, 24/SA, and 36/SA samples). In contrast, Figure 2b shows the synthesis with aluminum hydroxide (24/SH and 48/SH samples). The study using Al_2_O_3_ and Al(OH)_3_ was carried out to observe the behavior of other aluminum sources in the synthesis of SAPO-11. The prominent XRD peaks observed in the SAPO-11 phase with AEL structure are 2Θ = 8.0, 9.5, 20.4, 21.1, 22.3, 22.3, 22.8, and 23.3 degrees [38,39,40], as verified in the 12/SA, 24/SA and 36/SA samples, suggesting high crystallinity of the obtained SAPO-11 material. For sample 24/SA (Figure A1 in Appendix A), phase identification and refinement verified the presence of three phases, 77.51% SAPO-11 (AEL structure), 19.11% aluminum phosphate-tridymite, and 3.39% aluminum phosphate (V), also described as AlPO-5 (AFI structure). For 24/SH, around 36% SAPO-11 (AEL structure) and the remaining 64% aluminum phosphate (V) (AFI structure) were obtained (Figure A2 in Appendix A). In all samples, peaks were identified, referring to AEL structure (SAPO-11), and phases of AlPO_4_-Tridymite may are first be formed during the synthesis of SAPO-11 [33]. However, with shorter crystallization times, as in the 6/SA sample, the material formation begins, but longer crystallization times are required to complete the structure formation. One secondary phase was verified for both aluminum sources, with XRD peaks being identified as aluminum phosphate (V), AFI structure, (ICSD 88566). Moreover, the use of low crystalline structures of aluminum oxide that are more reactive leads to materials with more crystalline phases and that are less contaminated [33]. Another critical factor in the synthesis of these materials is the pH values. An increase in the pH values of the gel within the acid region leads to better materials. Thus, low pH values are ideal for aluminophosphate synthesis. The pH < 3 causes the appearance of dense phases, and pH > 10 causes a decrease in yield, owing to solubilization, and consequently leads to a lower crystallization degree [41]. Considering the pH for 24/SA and 24/SH samples, which presented as pH = 1 and 6 before crystallization and after crystallization at times of 6 and 10 h, respectively, the presence of secondary phases in the materials is explained.

Figure 2 shows the SEM images of the synthesized samples. The low magnification SEM image (Figure 2c) shows that the SAPO-11 molecular sieve exhibited a spherical structure, with a relatively smooth surface (Figure 2d), which is typical of materials obtained from colloidal silica stabilized in aqueous media [42,43]. Figure 2e, with low magnification, confirms the XRD patterns, showing the morphology of the 24/SH sample, the presence of the AEL structure, and small spherical particles characteristic of SAPO-11, and Figure 2f shows stick or plate-shaped particles with agglomerated crystals that usually are associated with some contamination, probably from AFI structure, as previously reported [34,43,44].

The yield and molar ratio of synthesized samples were calculated and are summarized in Table 3. For the obtained SA materials, it was observed that the yield increased with the crystallization time, whereas for SH samples, the crystallization time does not change the yield. Still, the secondary aluminum phosphate (V) phase (AFI structure) may be interfering in crystallization. To form the SAPO-11 structure, the molar ratios of P_2_O_5_/SiO_2_ = 3.33 and Si/Al = 0.15 are preferred. The SA and SH samples obtained show that the 24/SA presented the best conditions, being the best result for obtaining the AEL structure of SAPO-11.

**Figure 3:**
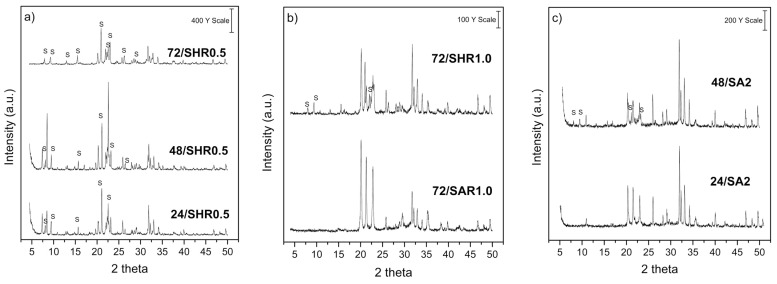

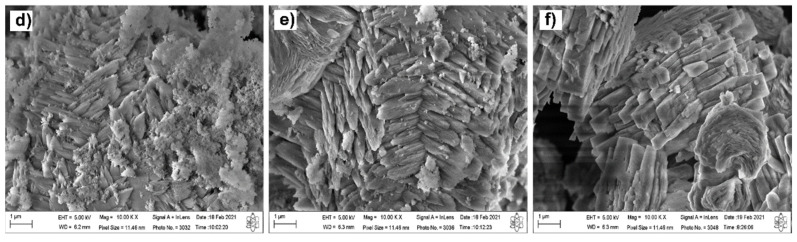
XRD patterns of samples by using phosphor powder (**a**–**c**) (S = SAPO-11) and SEM images of the 24/SHR0.5 (**d**), 48/SHR0.5 (**e**); 72/SHR0.5 (**f**).

**Figure A3:**
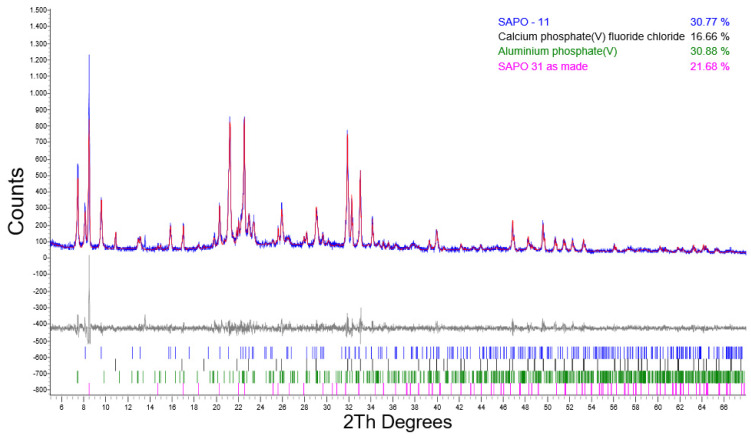
Rietveld refinement plot for 24/SHR0.5: the vertical lines provide the positions of all possible Bragg reflections for the aluminum phosphate (V) phases (database_code-ICSD 88566), calcium phosphate (V) fluoride chloride (database_code-ICSD 203026), structure of zeolite type AEL (database_IZA), and end structure of zeolite type ATO (database_IZA). The values of the standard agreement indices are Rp—10.74, Rwp—13.99, Rexp—10.77, GofF—1.30.4.

**Figure A4:**
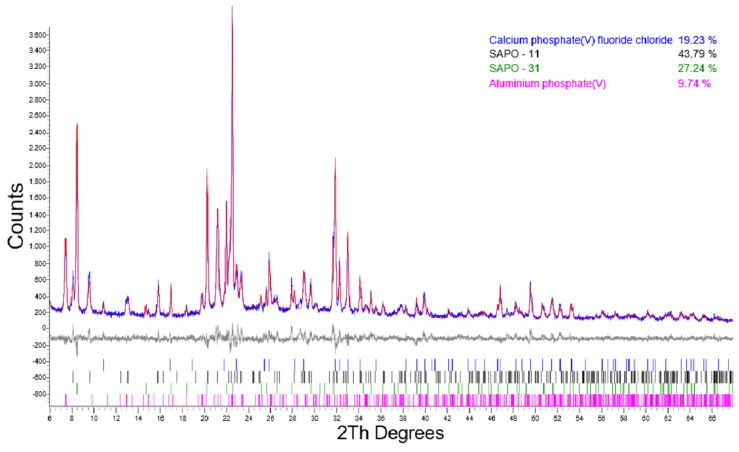
Rietveld refinement plot for 48/SHR0.5: the vertical lines provide the positions of all possible Bragg reflections for the aluminum phosphate (V) phases (database_code-ICSD 88566), calcium phosphate (V) fluoride chloride (database_code-ICSD 203026), structure of zeolite type AEL (database_IZA), and end structure of zeolite type ATO (database_IZA). The values of the standard agreement indices are Rp—7.86, Rwp—10.04, Rexp—6.23, GofF—1.61.

**Figure A5:**
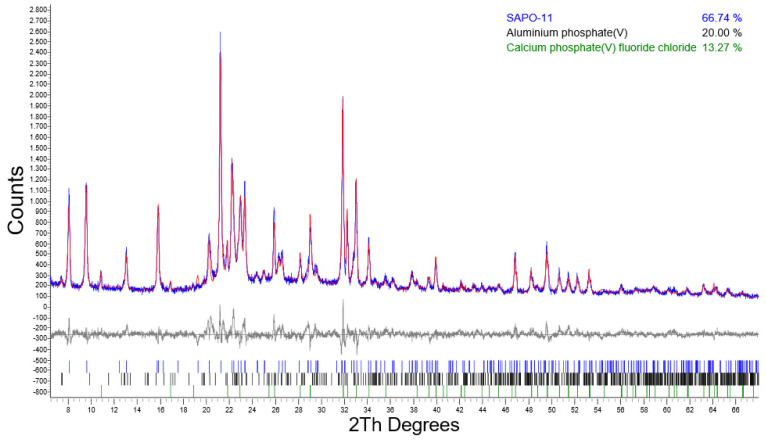
Rietveld refinement plot for 72/SHR0.5: the vertical lines provide the positions of all possible Bragg reflections for the aluminum phosphate (V) phases (database_code-ICSD 88566), calcium phosphate (V) fluoride chloride (database_code-ICSD 203026), and structure of zeolite type AEL (database_IZA). The values of the standard agreement indices are Rp—8.36, Rwp—10.77, Rexp—6.33, GofF—1.70.

The XRD patterns of SAPO-11 synthesized with the phosphor powder residue are presented in Figure 3. Figure 3a shows the samples synthesized with 0.5 g of residue and aluminum hydroxide as the additional aluminum source. The obtention of the AEL structure was proven by comparing the XRD pattern of the 24/SA standard sample. It was observed that there is a mixture of SAPO-11 phase (AEL structure) with calcium phosphate (V) fluoride chloride from the residue and aluminum phosphate (V) (AFI structure). However, in the synthesis times of 24 and 48 h, phases of SAPO-31 (ATO structure) were found (Figure A3, Figure A4 and Figure A5 in Appendix A). The percentages of the identified phases are presented in Table 4. The SAPO-11 phases (AEL structure) become more visible with the crystallization time, with 66.74% on sample 72/SH0.5. When the amount of residue was increased to 1.0 g (Figure 3b) or even 2.0 g (Figure 3c), the AEL structure was obtained from hydroxide and was not obtained when using aluminum oxide. From SEM, the morphologies identify the presence of spherical particles formed by cubic crystals and grouped plate-like crystallites often observed in SAPO-11 materials [45,46].

**Figure A6:**
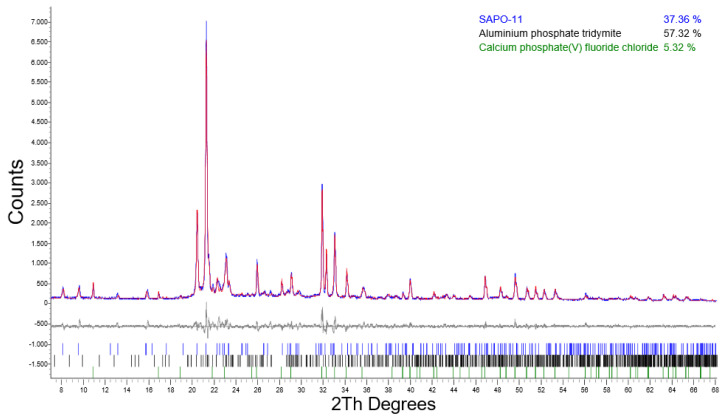
Rietveld refinement plot for 72/SHR1: the vertical lines provide the positions of all possible Bragg reflections for the aluminum phosphate tridymite phases, according to data presented in the literature [55], Calcium phosphate (V) fluoride chloride (database_code-ICSD 203026), and structure of zeolite type AEL (database_IZA). The values of the standard agreement indices are Rp—9.50, Rwp—12.28, Rexp—6.63, GofF—1.85.

**Figure A7:**
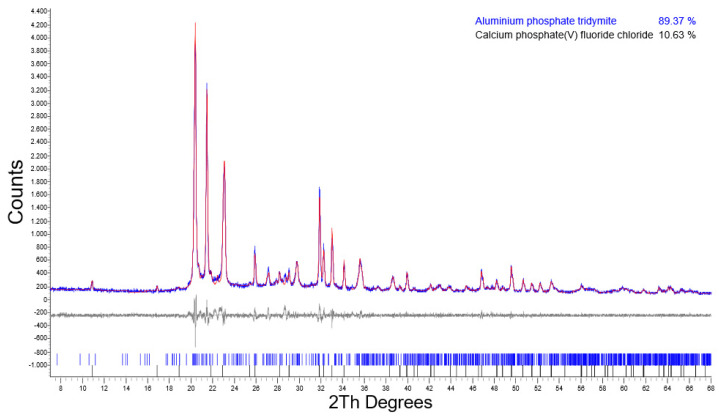
Rietveld refinement plot for 72/SAR1: the vertical lines provide the positions of all possible Bragg reflections for the aluminum phosphate tridymite phases, according to data presented in the literature [55]. Calcium phosphate (V) fluoride chloride (database_code-ICSD 203026). The values of the standard agreement indices are Rp—6.86, Rwp—8.94, Rexp—5.77, GofF—1.55.

For the 24/SRH0.5, 48/SRH0.5, and 72/SRH0.5 samples (Figure 3a), the SAPO-11 phase was in the range of 30–67%, with a good yield. Considering that these waste-based materials were obtained with a low amount of residue and molar ratios of P_2_O_5_/SiO_2_ = 3.8 and Si/Al = 0.11, as determined from XRF analysis, these conditions can thus be optimized to the formation of AEL structure (see Table 3). When the mass of residue was increased to 1.0 and 2.0 g in the gel, an increase in the pH from 4 to 10 was observed, however, without good crystallization and yield. This higher pH indicates that the alkaline media still prevails at the end of the crystallization process, even after hydrothermal treatment, evidencing a possible dissolution of unreacted silica present in the residue, consequently decreasing the crystallization of SAPO-11 materials containing more significant amounts of residue. The monitoring of the influence of pH on the synthesis of crystalline materials with AEL structure has been reported recently [47]. In synthesis 72/SHR1 only 37% of the SAPO-11 phase was formed, and in synthesis 72/SAR1 there was no formation of SAPO-11 or other phases of the SAPO type (Figure A6 and Figure A7 in Appendix A).

### 3.2. Specific Surface Area and Porositie

**Figure 4:**
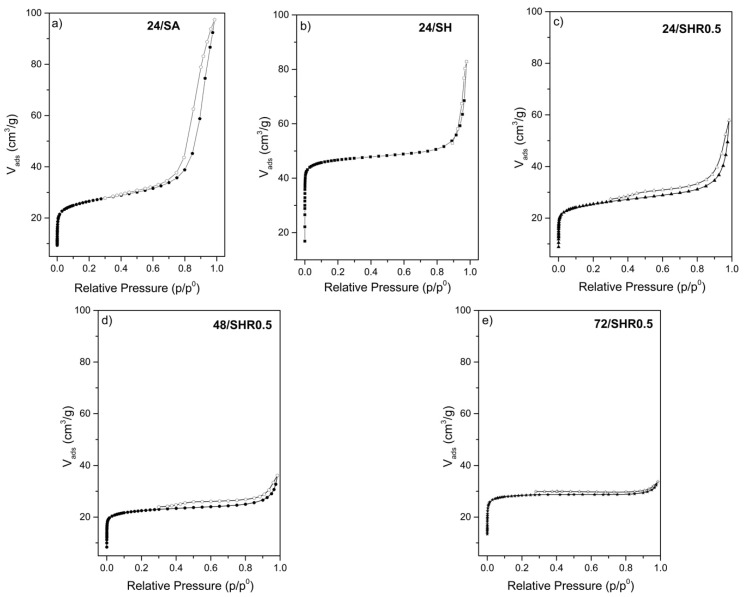
N_2_ sorption isotherms for standard SAPO-11 obtained: (**a**,**b**) from the conventional method; (**c**–**e**) samples and using phosphor residues.

N_2_ adsorption/desorption of samples is displayed in Figure 4. According to N_2_ adsorption/desorption isotherms, the 24/SA sample has Type I and H3-type hysteresis and more pronounced nitrogen adsorption at *p*/*p*_0_ of 0.2–0.6, indicating the presence of secondary mesoporosity [48,49].

For the 24/SH sample, a Type I isotherm was observed, with high adsorption of N_2_ at low relative pressures (*p*/*p*_0_ < 0.1), confirming the microporous nature with porosity [50], and present characteristics of materials with mesoporosity (Figure 4a,b). The micropore volume of sample 24/SH is 0.05 cm^3^/g and has a mesopore volume of 0.08 cm^3^/g. According to Table 5, the micropore and mesopore volumes of sample 24/SA are 0.02 cm^3^/g and 0.13 cm^3^/g, respectively. This relatively low volume of micropores is frequently reported [36,43]. The presence of hysteresis indicates that the volume between the particles of small crystals generates mesoporosity. The BET surface areas of samples 24/SA and 24/SH were 100 m^2^/g and 187 m^2^/g, respectively. In literature, the superficial area of SAPO-11 is in the range of 150 and 250 m^2^/g [51,52,53,54]. For the 24/SHR0.5, 48/SHR0.5, and 72/SHR0.5 samples, an H4 type hysteresis was observed, often found with aggregated crystals (Figure 4c). The surface areas, pore volumes of some SAPO-11 samples prepared by the conventional method, and residue use are given in Table 5.

According to the data summarized in Table 5, comparing the standard samples 24/SA and 24/SH, it is observed that the use of aluminum hydroxide (24/SH) led to an increase in the specific area of the material to 87 m^2^/g, which is almost double the value of the sample synthesized with alumina (24/SA), with an increase in the volume of micropores from 0.02 to 0.05 cm^3^/g. The large micropore volume and small hysteresis suggest a large fraction of zeolite crystals. As mentioned in Figure 2b and Table 4, the sample synthesized present AFI phase (64%), which resulted in greater pore opening [43]. This could contribute to greater adsorption of N_2_ and consequently greater specific area for the material.

When the phosphor powder residue was used as Al, Si, and P sources, with the addition of aluminum hydroxide in the gel, the specific area of the materials went from 87 to 113 m^2^/g. The increase in the crystallization time, from 24 h to 48 and 72 h, leads to more SAPO-11 phase; however, other phases are present too.

## 4. Conclusions

In this work, it was demonstrated that silicoaluminophosphate with AEL structure can be successfully obtained using a residue of fluorescent lamps. In summary, SAPO-11 molecular sieves have been synthesized by the hydrothermal method using dipropylamine as a template, by the conventional method, with and without residue of fluorescent lamp, and alumina oxide and aluminum hydroxide as Al source, changing the time and amount of residue in the gel composition. The materials characterization from XRD, SEM, and BET isotherms evidenced that the best results were obtained for 72 h of crystallization time. The aluminum hydroxide showed the best results, probably due to the presence of OH groups in the reactive hydrogel, increasing the pH for ca. 10, which is a value that increases the reactivity of the silicate media, promoting the formation of reactive silanol groups (Si-OH). After the addition of orthophosphoric acid, this pH decreases, and the lower pH enhances the activity of the phosphorus species favoring the polymerization of the aluminophosphate and silicate precursors, increasing the crystallization rate of the silicoaluminophosphate crystals. The best yield of the SAPO-11 material was obtained when a lower amount of residue was used for P_2_O_5_/SiO_2_ = 3.6 and Si/Al = 0.14; a yield of 75% was obtained, which was considered an excellent result. This sample was denoted as 72/SHR0.5. However, results obtained in 24 h were considered good too. Considering that less crystalline structures are more reactive, the greater the amount of residue used, the higher the difficulty in forming the desired structure and/or the greater the crystallization time required.

## Figures and Tables

**Table 1:** Parameters related to the aluminum source, synthesis time, and residue mass used in the synthesis of SAPO-11 materials and respective nomenclatures.

Sample	Al Source	Time (h)	Residue (g)
6/SA	Al_2_O_3_	6	None
12/SA	Al_2_O_3_	12	None
24/SA	Al_2_O_3_	24	None
36/SA	Al_2_O_3_	36	None
24/SH	Al(OH)_3_	24	None
48/SH	Al(OH)_3_	48	None
24/SHR0.5	Al(OH)_3_	24	0.5
48/SHR0.5	Al(OH)_3_	48	0.5
72/SHR0.5	Al(OH)_3_	72	0.5
72/SAR1	Al_2_O_3_	72	1.0
72/SHR1	Al(OH)_3_	72	1.0
24/SAR2	Al_2_O_3_	24	2.0
48/SAR2	Al_2_O_3_	48	2.0

**Table 2:** Chemical composition from the residue of the fluorescent lamp.

Composition	Quantities (%)
CaO	48.71
P_2_O_5_	27.7
SiO_2_	9.98
Al_2_O_3_	8.9
MgO	1.7
Cl	0.83
Fe_2_O_3_	0.55
K_2_O	0.48
SrO	0.34
Sb_2_O_3_	0.3
MnO	0.24
TiO_2_	0.1
V_2_O_5_	0.05
PbO	0.05

**Table 3:** Molar ratios and yield for the materials.

Samples	Molar Ratio		Yield ^†^ (%)
	P_2_O_5_/SiO_2_	Si/Al	
Residue	2.35	0.51	-
6/SA	1.8	0.23	-
12/SA	1.08	0.23	55
24/SA	1.93	0.16	68
36/SA	2.08	0.12	72
24/SH	1.85	0.17	41
48/SH	1.65	0.17	38
24/SHR0.5	3.8	0.11	63
48/SHR0.5	3.8	0.13	64
72/SHR0.5	3.6	0.14	75
72/SHR1	3.33	0.22	n.d.
24/SAR2	2.14	0.30	n.d.
48/SAR2	2.41	0.27	n.d.

^†^ Yield calculated in dry base, considering the quantities of oxides (silicon, aluminum, sodium, and phosphorus) added to the synthesis gel and the solid product recovered after drying and filtering.

**Table 4:** Identified Phases by Rietveld refinement.

Samples	Identified Phases (%)				
	SAPO-11	SAPO-31	Calcium Phosphate (V) Fluoride Chloride	Aluminum Phosphate (V)	Aluminum Phosphate Tridymite
24/SA	77.51	n.d.	n.d.	3.39	19.10
24/SH	35.39	n.d.	n.d.	64.61	n.d.
24/SHR0.5	30.77	21.68	16.66	30.88	n.d.
48/SHR0.5	43.79	27.24	19.23	9.74	n.d.
72/SHR0.5	66.74	n.d.	13.27	20.00	n.d.
72/SHR1	37.36	n.d.	5.32	n.d.	57.32
72/SAR1	n.d.	n.d.	10.63	n.d.	89.37

**Table 5:** Textural parameters result obtained from N_2_ adsorption and desorption.

SAPO-11	BET	t-Plot Harkins-Jura-de-Boer		Gurvich VTP
Samples	SBET (m^2^/g)	Vo (cm^3^/g)	St (m^2^/g)	(cm^3^/g)
24/SA	100	0.02	50	0.15
24/SH	187	0.05	56	0.13
24/SHR0.5	97	0.03	20	0.08
48/SHR0.5	87	0.02	31	0.06
72/SHR0.5	113	0.03	26	0.05

## Data Availability

Not applicable.

## Sample Availability

Samples of the compounds are available from the authors.
